# Enhanced p53 Levels Are Involved in the Reduced Mineralization Capacity of Osteoblasts Derived from Shwachman–Diamond Syndrome Subjects

**DOI:** 10.3390/ijms222413331

**Published:** 2021-12-11

**Authors:** Annalisa Frattini, Simona Bolamperti, Roberto Valli, Marco Cipolli, Rita Maria Pinto, Elena Bergami, Maria Rita Frau, Simone Cesaro, Michela Signo, Valentino Bezzerri, Giovanni Porta, Abdul Waheed Khan, Alessandro Rubinacci, Isabella Villa

**Affiliations:** 1 Institute for Genetic and Biomedical Research (IRGB), UOS Milano CNR, Via Fantoli, 15/16, 20138 Milano, Italy; 2 Department of Medicine and Surgery (DMC), Universita’ degli Studi dell’Insubria, Via J.H. Dunant, 5, 21100 Varese, Italy; roberto.valli@uninsubria.it (R.V.); giovanni.porta@uninsubria.it (G.P.); khan_ibge@yahoo.com (A.W.K.); 3 Bone Metabolism Unit, IRCCS Ospedale San Raffaele, Via Olgettina, 60, 20132 Milano, Italy; bolamperti.simona@hsr.it (S.B.); michela.signo@studenti.unipd.it (M.S.); rubinacci.alessandro@hsr.it (A.R.); villa.isabella@hsr.it (I.V.); 4 Cystic Fibrosis Center, Azienda Ospedaliera Universitaria Integrata di Verona, Piazzale Aristide Stefani, 1, 37126 Verona, Italy; marco.cipolli@aovr.veneto.it; 5 Department of Onco-Hematology, Ospedale Bambino Gesù IRCCS, Piazza S.Onofrio, 4, 00165 Roma, Italy; ritamaria.pinto@opbg.net; 6 Pediatric Onco-Hematology, IRCCS Policlinico San Matteo, Viale Camillo Golgi, 19, 27100 Pavia, Italy; e.bergami@smatteo.pv.it; 7 Pediatrics and Intensive Neonatal Therapy, Ospedale San Francesco, Via Salvatore Mannironi, 08100 Nuoro, Italy; mrfrau@aslnuoro.it; 8 Pediatric Hematology Oncology, Ospedale Donna Bambino, Azienda Ospedaliera Universitaria Integrata, Piazzale Aristide Stefani, 1, 37126 Verona, Italy; simone.cesaro@aovr.veneto.it; 9 Cystic Fibrosis Center, Azienda Ospedaliero Universitaria Ospedali Riuniti di Ancona, Via Conca, 71, 60126 Ancona, Italy; valentino.bezzerri@gmail.com

**Keywords:** bone cells, mineralization, p53, transcriptome, ribosomopathies

## Abstract

Shwachman–Diamond syndrome (SDS) is a rare autosomal recessive disorder characterized by bone marrow failure, exocrine pancreatic insufficiency, and skeletal abnormalities, caused by loss-of-function mutations in the *SBDS* gene, a factor involved in ribosome biogenesis. By analyzing osteoblasts from SDS patients (SDS-OBs), we show that SDS-OBs displayed reduced *SBDS* gene expression and reduced/undetectable SBDS protein compared to osteoblasts from healthy subjects (H-OBs). SDS-OBs cultured in an osteogenic medium displayed a lower mineralization capacity compared to H-OBs. Whole transcriptome analysis showed significant differences in the gene expression of SDS-OBs vs. H-OBs, particularly in the ossification pathway. SDS-OBs expressed lower levels of the main genes responsible for osteoblastogenesis. Of all downregulated genes, Western blot analyses confirmed lower levels of alkaline phosphatase and collagen type I in SDS-OBs than in H-OBs. Interestingly, SDS-OBs showed higher protein levels of p53, an inhibitor of osteogenesis, compared to H-OBs. Silencing of *Tp53* was associated with higher collagen type I and alkaline phosphatase protein levels and an increase in SDS-OB mineralization capacity. In conclusion, our results show that the reduced capacity of SDS-OBs to mineralize is mediated, at least in part, by the high levels of p53 and highlight an important role of SBDS in osteoblast functions.

## 1. Introduction

Shwachman–Diamond syndrome (SDS1, OMIM #260400) is a rare autosomal recessive monogenic inherited multisystemic disorder [1,2] mainly characterized by exocrine pancreatic dysfunction, bone marrow failure associated with specific chromosome instability [3], and predisposition toward myelodysplasia syndrome (MDS) or acute myeloid leukemia (AML) [4,5]. The estimated incidence of SDS is 1/75,000. However, it is difficult to determine the real frequency of SDS in the general population due to its high phenotypic variability [6]. Approximately 90% of patients affected by SDS carry biallelic mutations in the Shwachman–Bodian–Diamond Syndrome (*SBDS*) gene, which is located on chromosome 7q11.21 [7]. The most frequent biallelic inactivating mutations in the *SBDS* gene are 183-184TA > CT and 258 + 2T > C, both originating from gene conversion between *SBDS* and its pseudogene (*SBDSP1*) located, in an inverted orientation, at 5.8 Mb distally [8,9]. The first mutation introduces a premature in-frame termination codon, resulting in the amino acid change K62X. The mutation 258 + 2T > C affects the donor splice site of intron 2, promoting the use of an upstream cryptic donor splice site at position 251_252, which generates a transcript with an 8 bp deletion, resulting in a frameshift and a premature termination codon (C84fsX3). The presence of truncated proteins due to these mutations has not been reported. No homozygotes for the null allele (i.e., c.183_184TA > CT) have been reported in humans, suggesting that the complete loss of the functional SBDS protein is incompatible with life. This finding is in agreement with other evidence obtained in Dictyostelium discoideum [10], in zebrafish [11], and in mice, in which the complete loss of *Sbds* results in early embryonic lethality [12]. Therefore, this implies that SDS patients should express at least one hypomorphic *SBDS* allele, as shown by the detection, although at a very low level, of the full-length SBDS protein in their cells [9,10].

Beside the reported role of the SBDS protein in the spindle apparatus’s stability and chromosome segregation [13,14], in DNA replication and repair [15], in telomere protection [16], and in endoplasmic reticulum stress, SBDS plays an essential role in the final step of ribosomal maturation [17]. The SBDS protein, through the coupling with EFL1 (GTPase Elongation Factor-like 1), promotes the release of eIF6 (Eucaryotic Initiation Factor 6) from the pre-60S ribosomal subunit to generate the mature 80S functional ribosome [18,19]. The association of the large 60S ribosomal subunit with the small 40S subunit is sterically blocked by eIF6. The release of eIF6 allows the joining of the 60S and 40S subunits and the formation of the translationally active 80S ribosome [4,20]. In addition, SBDS plays a role in the conformational maturation of the ribosomal P-site [19].

A variety of skeletal abnormalities, evolving over time, are commonly reported in patients with SDS and are related to abnormal development of the growth plates, delay of secondary ossification centers, resulting in metaphyseal dysostosis, particularly at the femoral head, shortened ribs with flared anterior ends, and costochondral thickening [21,22]. The skeletal dysplasia is highly variable, even in patients with identical genotypes, and its severity and location vary with age [23]. Remarkably, the bone of the majority of SDS-affected individuals, beside its dysplastic morphology, shows the features of low-turnover osteoporosis with an increased risk of fragility fracture: a tragic outcome in individuals already affected by severe deterioration in their quality of life. The reduction in bone mass is present at different skeletal sites including the ribs, femurs, knees, heads of humeri, wrists, ankles, and vertebrae [21,24,25]. A reduced trabecular bone volume (BV/TV), low numbers of osteoblasts (OB.S/BS) and osteoclasts (OCs/BS), and a reduced amount of osteoid (OS/BS) were also reported in the histomorphometric analysis of the few bone biopsies obtained in one study [23]. In particular, the observed low values of BV/TV, OB.S/BS, OS/BS, and OCs/BS consistently indicated a focal bone remodeling unbalance, primarily related to the inefficiency of the bone formation phase. Mesenchymal stem cells (MSCs) of SDS patients had morphology, growth kinetics, and expression of surface markers similar to normal MSCs and were able to differentiate into adipogenic, osteogenic, and chondrogenic lineages [26], suggesting that they are not involved in the inefficient bone formation observed in SDS.

Osteoblasts are characterized by high protein secretion, particularly structural proteins, such as collagen, that are critical for bone mechanical competence [27]. It is therefore likely that the altered SBDS protein levels, which affect global protein translation, might induce defective translation of critical proteins in osteoblasts that are involved in the acquisition and maintenance of bone mass and quality during skeletal development [28].

Considering the bone feature defects observed in SDS, we designed the present study to characterize the role of *SBDS* in osteoblasts’ functionality. For this purpose, we derived osteoblasts directly from the bone of SDS patients (SDS-OBS) and examined their gene expression profile and their ability to mineralize compared to osteoblasts from healthy subjects (H-OBs). The availability of osteoblasts from a monogenic disorder offers the unique opportunity to assess the effects of a single gene mutation on the physiology of bone metabolism and enables the identification of novel molecular mechanisms, as has occurred for other monogenic disorders [29]. At present, this is the first study to evaluate the functions of osteoblasts derived from SDS patients.

## 2. Results

### 2.1. Low SBDS Levels in Osteoblasts Derived from SDS Patients

We succeeded in establishing osteoblast primary cultures from remnants of patients’ bone biopsies, which are performed yearly in the context of their routinely medical check-up for myeloid transformation. In basal culture conditions, osteoblasts derived from these patients (SDS-OBs) showed a similar morphology, proliferation, and apoptosis (Figure S1) compared to osteoblasts derived from healthy subjects (H-OBs).

Considering that SDS patients should express at least one hypomorphic *SBDS* allele, as shown by the detection of a very low level of the full-length SBDS protein in their cells [9,10], we investigated the presence of the residual SBDS wild-type transcript. Total RNA was extracted from lymphoblastoid cell lines and osteoblast cultures of a healthy control (H), a patient, SDS3, who carries the mutation c.258 + 2T > C in the heterozygous state, and a patient, SDS9, who carries the mutation in the homozygous state (Table 1).

**Figure 1:**
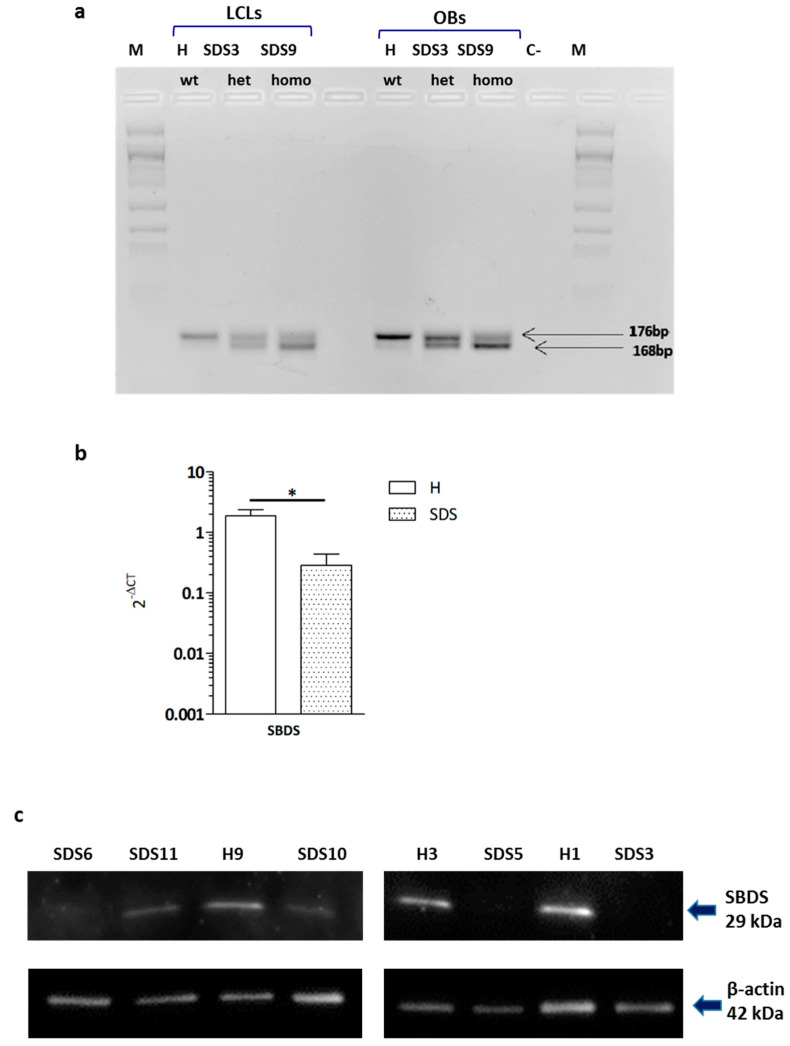

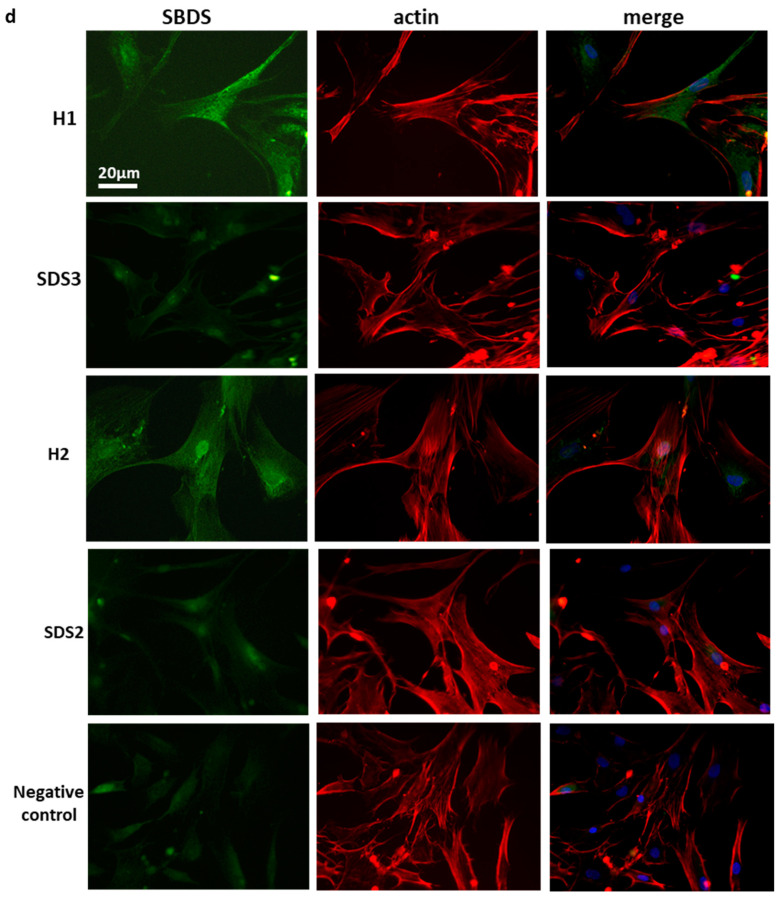
Osteoblasts derived from Shwachman–Diamond syndrome subjects. (**a**) RT-PCR on RNA of lymphoblastoid cells (LCLs) and osteoblasts (OBs) derived from a healthy control (H), a patient (SDS3) who carries the mutation c.258 + 2T > C in the heterozygous state, and a patient (SDS9) who carries the mutation in the homozygous state. In both patients, there is a residual transcript of wt *SBDS*. To detect the residual wt amplicon, cDNA of the patients was used at a concentration 2 times (SDS3) and 10 times (SDS9) higher than the cDNA concentration of the healthy subject. C-, negative control; M, marker 100 bp. (**b**) Relative mRNA expression of *SBDS* in osteoblasts from SDS patients (SDS, *n* = 6) and age- and sex-matched healthy subjects (H, *n* = 6). Mann–Whitney test, * *p* < 0.05 vs. healthy. (**c**) Representative image of Western blot analyses of SBDS protein expression in osteoblasts from healthy subjects (H3, H1) and SDS patients (SDS5, SDS3). (**d**) Representative image of immunofluorescence for SBDS in osteoblasts derived from two healthy subjects (H1, H2) and two SDS patients (SDS3, SDS2) matched by age and sex. SBDS, green; actin fibers, red; negative control: secondary antibody; magnification 40×, scale bar: 20 μm.

As expected, in the healthy controls, PCR analysis showed a single band of 176 base pairs, which corresponds to the full-length *SBDS* transcript. SDS3 (het) displayed the band of 176 base pairs and a second band of 168 base pairs which corresponds to the truncated transcript of *SBDS*. SDS9 (homo) had the same bands as SDS3, with the full transcript band being less intense than the truncated transcript band (Figure 1a). Noteworthy, to detect any visible band in the patient-derived cells, an amount of cDNA 2 times higher for patient SDS3 and 10 times higher for patient SDS9 than for cells obtained from the healthy subject was necessary. The presence of low levels of the normal *SBDS* transcript (verified by sequencing) suggests that the mutation 258 + 2T > C causes the conversion of the nucleotide T > C, creating a new non-canonical GC 5′ splice site (Figure S2) that allows the transcription of the correct form of *SBDS* mRNA [30]. The results obtained in two different cell lines from SDS patients confirm that the mutation 258 + 2T > C allows a hypomorphic expression of the wild-type *SBDS* allele. We then evaluated the total *SBDS* transcript and found that it was significantly (*p* < 0.001) lower in SDS-OBs compared to H-OBs (Figure 1b).

The SBDS protein amount analyzed by Western blot in SDS-OBs and compared to H-OBs showed lower or almost undetectable quantities in SDS-OBs (Figure 1c). This observation was confirmed by immunofluorescence staining (Figure 1d).

### 2.2. Mineralization of SDS-OBs Is Impaired

**Figure 2:**
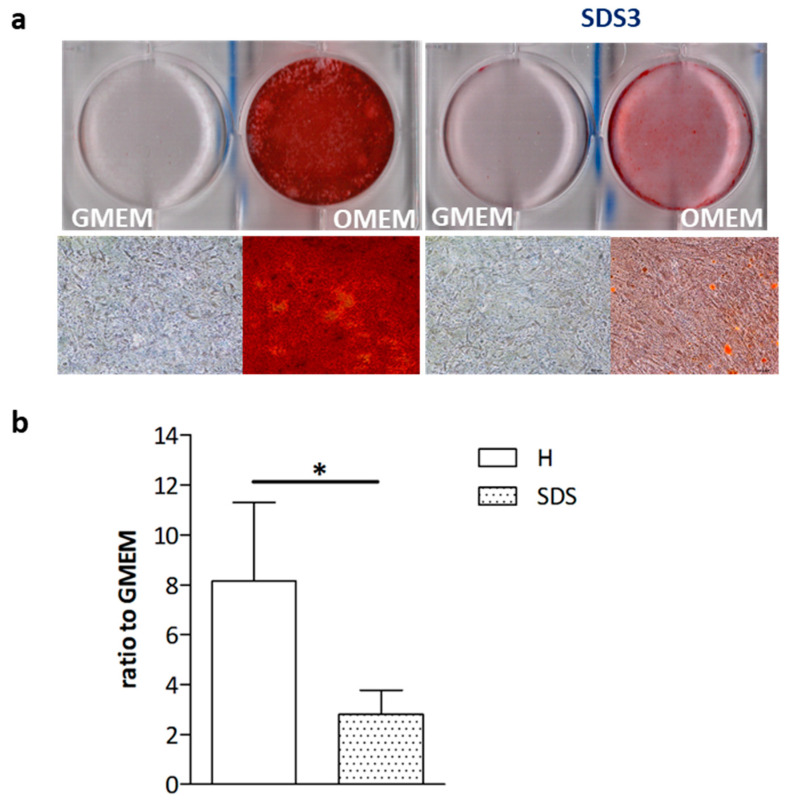
Mineralization of SDS-OBs and H-OBs. (**a**) Representative image of Alizarin Red staining of osteoblasts from a healthy subject (H1) and an SDS patient (SDS3), matched by age and sex, cultured for 28 days in growth medium (GMEM) or osteogenic medium (OMEM), and (**b**) relative quantification (H, *n* = 6; SDS, *n* = 9). Mann–Whitney test, * *p* < 0.05.

In order to evaluate SDS-OBs’ functionality, we analyzed the capacity of SDS-OBs to mineralize. Both SDS-OBs and H-OBs were cultured for 28 days in a differentiation medium to induce mineralization (OMEM). Quantification of the absorbance of Alizarin Red staining showed a reduced mineral deposit in the SDS-OB culture compared to the H-OB culture, which was statistically significant (*p* < 0.05; Figure 2).

### 2.3. Osteogenesis-Related Gene and Relative Protein Expressions Are Reduced in SDS-OBs

**Figure 3:**
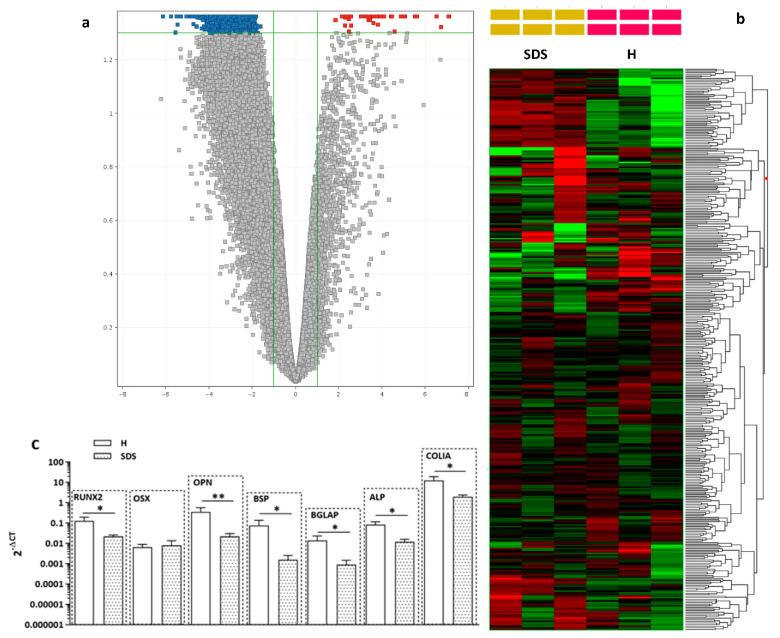
Gene expression in SDS-OBs. (**a**) Volcano plot of whole gene expression of SDS-derived osteoblasts vs. healthy subject-derived osteoblasts. The x-axis displays the log2 fold change value, and the y-axis corresponds to the negative logarithm of the base 10 of the *t*-test *p*-values. A total of 2373 genes were differentially expressed in SDS-derived osteoblasts vs. healthy osteoblasts. The red dots represent upregulated genes (43 genes), and the blue dots represent downregulated genes (2330 genes). The gray dots correspond to genes with no significant difference. (**b**) Heatmap showing differentially expressed mRNAs of Gene Ontology ossification (GO:0001503) comparing 3 healthy individuals vs. 3 patients with SDS (SDS1, SDS5, and SDS11). Each row represents one mRNA, and each column represents a sample. Red, upregulation; green, downregulation. (**c**) Relative mRNA expression of the main osteogenesis-related genes in osteoblasts of healthy subjects (H; *n* = 8) and SDS patients (SDS; *n* = 10). Runt-related transcription factor 2 (*Runx2*), osterix (*OSX*), osteopontin (*OPN*), bone sialoprotein (*BSP*), osteocalcin (*BGLAP*), alkaline phosphatase (*ALP*), collagen type I (*COL1A*). Mann–Whitney test, * *p* < 0.05; ** *p* < 0.01.

Considering the impaired mineralization capacity of SDS-OBs, we evaluated the SDS-OB transcriptome by microarray analysis. Gene expression profile analysis showed a generalized reduction in gene expression in SDS-OBs vs. H-OBs. The volcano plot analysis of the FC > 2 entries conducted with a moderated *t*-test and Benjamini–Hochberg correction (corrected *p*-value < 0.05) revealed 2330 downregulated entries and only 43 upregulated entries (Figure 3A). The heatmap of genes involved in the ossification process (GO:0001503, 308 genes in the *Homo sapiens* taxon) with FC > 2 (Figure 3B) showed differences between SDS-OBs and H-OBs in a limited number of genes, with 80 downregulated genes and 49 upregulated genes (Table S1) (raw data are available at https://www.ebi.ac.uk/arrayexpress/experiments/E-MTAB-9397; 24 July 2020).

Further analysis with quantitative PCR (RT-qPCR) of the main osteogenesis-related genes showed a lower expression of runt-related transcription factor 2 (*Runx2*; *p* < 0.05), osteopontin (*OPN*; *p* < 0.01), bone sialoprotein (*BSP*; *p* < 0.05), osteocalcin (*BGLAP*; *p* < 0.05), alkaline phosphatase (*ALP*; *p* < 0.05), and collagen type I (*COL1A1*; *p* < 0.05) in SDS-OBs compared to H-OBs, while the expression levels of osterix (*OSX*) were unchanged (Figure 3C).

**Figure 4:**
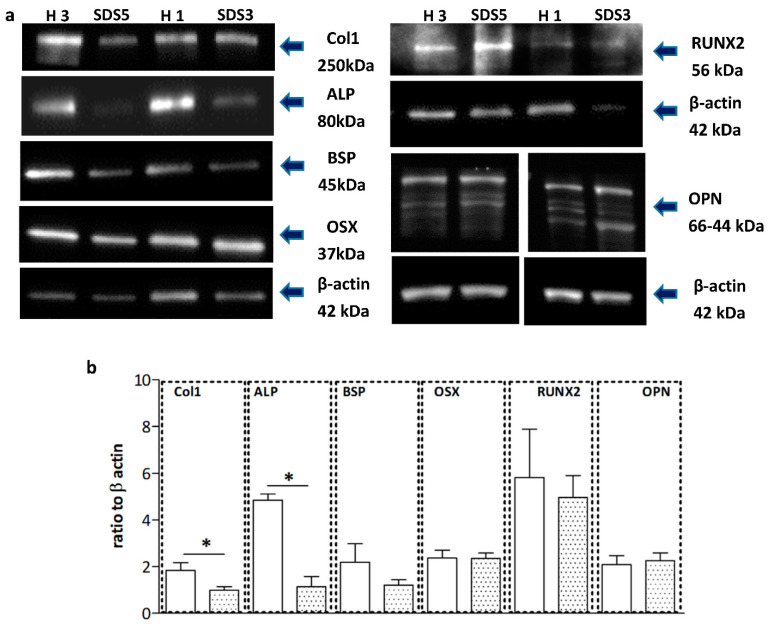
Protein expression in SDS-OBs. (**a**) Representative image of Western blot analyses of collagen type I (Col1), alkaline phosphatase (ALP), bone sialoprotein (BSP), runt-related transcription factor 2 (RUNX2), and osterix (OSX) protein expression in osteoblasts from healthy subjects (H3, H1) and SDS patients (SDS5, SDS3), and (**b**) relevant quantification (H; *n* = 5; SDS; *n* = 9). Mann–Whitney test, * *p* < 0.05.

Protein levels of ALP and collagen type I analyzed by Western blot were significantly (*p* < 0.05) lower in SDS-OBs in respect to H-OBs, whereas RUNX2, OSX, and OPN showed no difference between the two groups. BSP showed a trend of reduction, although not reaching statistical significance (Figure 4a,b).

### 2.4. High p53 Levels Are Involved in SDS-OB Functional Impairment

**Figure 5:**
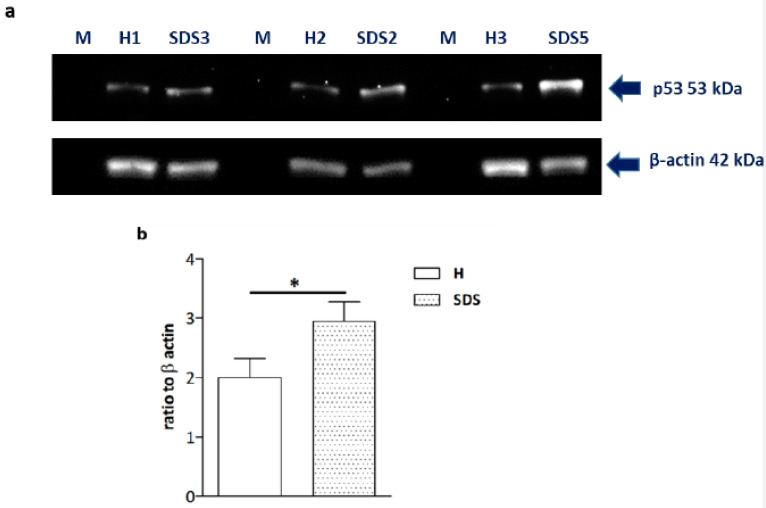
High p53 expression in SDS-OBs. (**a**) Representative image of Western blot analyses of p53 protein expression in osteoblasts from age- and sex-matched healthy subjects (H1, 2, 3) and SDS patients (SDS3, 2, 5), and (**b**) relevant quantification (*n* = 9). Mann–Whitney test, * *p* < 0.05.

Since previous studies have shown that bone marrow from SDS patients expresses higher levels of p53 than that from healthy subjects [31], and that p53 exerts an inhibitory action on osteogenesis [32], we evaluated the p53 protein amount in SDS-OBs by Western blot (Figure 5a). Our results show that the p53 content in SDS-OBs was significantly higher (*p* < 0.05) compared to that in H-OBs (Figure 5b). This increase could not be ascribed to activating mutations of the *Tp53* gene, as the sequencing of the coding region of *Tp53* performed on RNA from SDS-OBs (*n* = 11) and H-OBs (*n* = 4) only showed the presence of the missense SNP rs1042522 in all the subjects, which occurs in the worldwide population [33].

**Figure 6:**
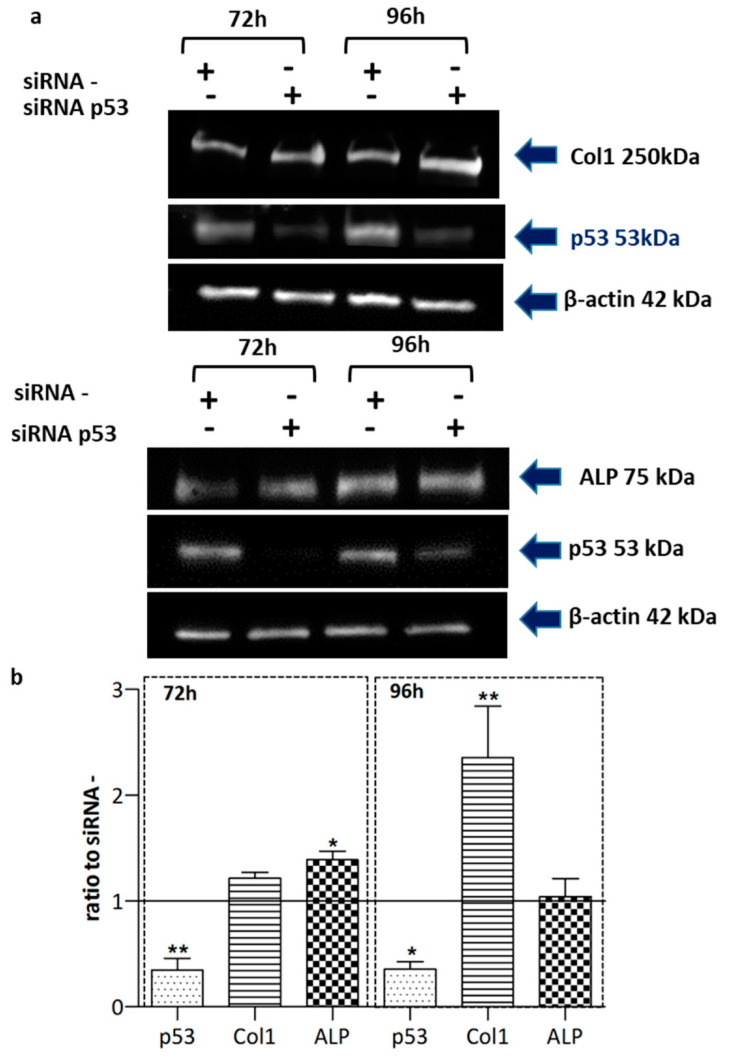
Silencing of p53 in SDS-OBs. (**a**) Representative image of Western blot analyses of collagen type I (Col1), ALP, and p53 in osteoblasts from an SDS patient (SDS13) treated for 72 h and 96 h with p53 siRNA (5nM) or siRNA negative control (siRNA-), and (**b**) relevant quantification (*n* = 5). Friedman test, * *p* < 0.05; ** *p* < 0.01.

To find the possible link between p53 and collagen and ALP, we treated SDS-OBs with a small interference RNA (siRNA, 5nM) targeting *Tp53*. The treatment induced a significant (*p* < 0.05) reduction in p53 protein levels after 72 h and 96 h. Concurrently, ALP increased at 72 h (*p* < 0.05) and collagen type 1 protein increased significantly at 96 h (*p* < 0.01) (Figure 6a,b), suggesting that p53 is involved in the lower levels of these proteins observed in SDS-OBs.

**Figure 7:**
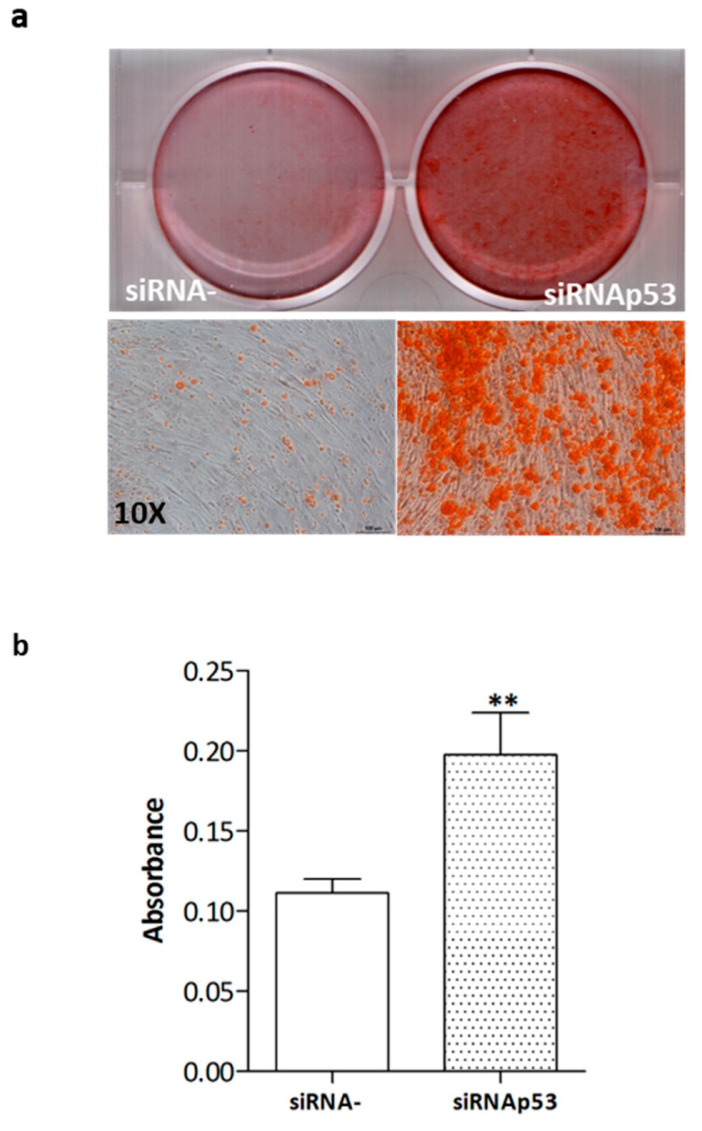
Mineralization in siRNAp53-treated SDS-OBs. (**a**) Representative image of Alizarin Red staining of osteoblasts from an SDS patient (SDS12), cultured for 28 days in osteogenic medium and treated twice a week with p53 siRNA (5 nM) or siRNA negative control (siRNA-), and (**b**) relevant quantification (*n* = 3, paired Student’s *t*-test, ** *p* < 0.01).

To evaluate if p53 was involved in the reduced mineralization capacity of SDS-OBs, the cells were cultured in an osteogenic medium for 28 days and treated with the *Tp53* siRNA twice a week. The silencing of p53 was able to induce a significant increase in SDS-OBs’ mineral deposition compared to that of siRNA negative control-treated SDS-OBs, as shown by Alizarin Red staining (Figure 7a,b).

## 3. Discussion

The present study shows that low levels of the SBDS protein negatively affect osteoblast mineralization capacity and compromise the physiological expression of osteogenesis-related genes in osteoblasts, thus supporting the hypothesis that low SBDS levels affect the osteogenic process, likely contributing to the altered bone phenotypic traits of SDS.

Besides low/undetectable SBDS protein amounts, SDS-OBs displayed low levels of *SBDS* mRNA, possibly due to the activation of the nonsense-mediated mRNA decay (NMD) process. The NMD machinery selectively recognizes and degrades mRNAs whose open reading frame is truncated by the presence of a premature stop codon, in order to protect the cell from the accumulation of C-terminal truncated proteins with potential deleterious functions [34]. In fact, in SDS, the mutations 183-184TA > CT and 258 + 2T > C elicit a premature stop codon that could generate truncated forms of SBDS which are possibly detrimental for the cell.

The analysis of the whole transcriptome showed a downregulation in the overall gene expression, suggesting a global reduction in osteoblast activities. In particular, the expression of the main genes related to osteogenesis, except for *OSX*, was significantly reduced in SDS-OBs. The reduction in the gene expression of *RUNX2* and *OPN* was not associated with a significant reduction in protein content. RUNX2 and OSX are transcription factors essential for osteoblast differentiation. RUNX2 induces the expression of the main bone matrix proteins during the early phases of osteoblast differentiation, but it is not required to maintain their expressions in mature osteoblasts. OSX further fosters mesenchymal cells’ differentiation to osteoblasts [35]. It is not surprising to observe any difference in these transcription factors between SDS-OBs and H-OBs as the cells obtained from trabecular bone specimens are already committed and RUNX2 and OSX do not play a fundamental role in the maintenance of mature osteoblast activities [35].

Bone matrix components such as BSP, ALP, and collagen type I showed a lower protein expression in SDS-OBs. BSP and ALP expressions are tightly associated with the mineralization process and mark the early stage of mineralization. As with the major glycoproteins of the bone matrix, BSP contains the tripeptide sequence Arg-Gly-Asp (RGD) that mediates the interaction between the matrix and the integrins of bone cells. Moreover, BSP has a high capacity of binding calcium ions and is a determining factor in promoting the nucleation of hydroxyapatite crystals in a variety of in vitro assays [36]. The *Bsp* knockout mouse model is associated with defects in bone mineralization and formation [37]. Due to its involvement in the regulation of matrix mineralization and in the early stage of osteogenesis, a low expression of *BSP* could contribute, together with the above-mentioned proteins, to the impaired mineralization observed in SDS-OBs. Alkaline phosphatase is a ubiquitous membrane-bound glycoprotein that plays an important role in matrix mineralization. It catalyzes the hydrolysis of phosphate monoesters at basic pH values. By favoring the ratio between inorganic phosphate (*Pi*), which fosters mineralization, and inorganic pyrophosphate (*PPi*), which instead inhibits this process, ALP exerts a primary role in the regulation of mineralization. The reduced expression of ALP might conversely favor the reduction in the Pi/PPi ratio, thus leading towards impaired mineralization [38]. Since the non-collagenous matrix proteins contribute to the correct development of the skeleton as well as the acquisition and maintenance of bone strength, it is likely that their lower levels could contribute to the bone phenotype of SDS patients.

Collagen type I constitutes the matrix scaffold that will be mineralized in a following step. Therefore, the lower collagen amount produced by SDS-OBs could contribute to a lower matrix deposition. As collagen confers mechanical stiffness and strength to bone, the lower amount of collagen produced by SDS-OBs could lead to an enhanced fracture risk in SDS patients and is in agreement with the lower amount of osteoid observed in bone biopsies of these patients [23].

As previously reported in the bone marrow of SDS patients [31], SDS-OBs also express a higher amount of the tumor suppressor protein p53. p53 is known to increase after DNA damage [39] and/or upon ribosomal biogenesis impairment [40], as occurs in SDS [4]. A previous study has shown that p53 has an inhibitory action on osteogenesis [41].

The evidence that the high levels of p53 are involved in the impaired osteogenic process of SDS-OBs was obtained by silencing *Tp53* in SDS-OBs. The silencing of *Tp53* was able to rescue collagen type 1 and alkaline phosphatase levels, and mineralization. *Tp53*siRNA-treated SDS-OBs actually displayed a higher mineralization capacity compared to SDS-OBs treated with a negative scramble control. This result outlines the inhibiting role of p53 in the mineralization process of osteoblasts and is in agreement with previous results demonstrating that, in the absence of p53, as in *p53*-deficient mice, bone nodule formation and alkaline phosphatase staining were accelerated [32].

In conclusion, this study demonstrates that the lack of SBDS in osteoblasts is associated with high levels of p53 that are involved in the reduced mineral deposition of SDS osteoblasts. These findings are coherent with the low-bone mass phenotype observed in SDS patients and provide evidence that SBDS plays an important role in osteoblast functions.

## 4. Materials and Methods

### 4.1. Cell Culture

Human samples were obtained and analyzed in accordance with the Declaration of Helsinki, after written consent. All protocols were approved by the Azienda Ospedaliera Universitaria Integrata (Verona, Italy), approval No. 658 CESC, and Azienda Ospedaliero Universitaria Ospedali Riuniti (Ancona, Italy), approval No. CERM 2018-82. Primary human osteoblast cultures were established by means of a modified version of the Gehron-Robey and Termine [42] procedure from bone remnants of bone marrow biopsies of SDS patients, or from waste material during orthopedic surgery or bone marrow biopsies of healthy controls. The trabecular bone was cut into small pieces and incubated with rotation at 37 °C for 30 min with Joklik’s modified MEM (Sigma-Aldrich, Milan, Italy) serum-free medium containing 0.5 mg/mL type IV collagenase (Sigma-Aldrich, Milan, Italy). The pieces from each sample were then placed in 25 cm^2^ flasks and cultured in Iscove’s modified medium (IMDM, Sigma-Aldrich, Milan, Italy) containing 10% FBS (Euroclone srl, Milan, Italy), penicillin, streptomycin, and amphotericin B (Sigma-Aldrich, Milan, Italy, 1:100) until confluence; the cells were maintained in an incubator at 37 °C and at a humidified atmosphere of 5% CO2. The culture medium was changed every 2–3 days. Cells were used at the second passage to reduce the possibility of phenotype changes. To induce mineralization, confluent osteoblasts were cultured for 28 days in osteogenic medium containing 10^−7^ M dexamethasone (Sigma-Aldrich, Milan, Italy), 50 ng/mL ascorbic acid (Sigma-Aldrich, Milan, Italy), and 5 mM β-glicerophosphate (Sigma-Aldrich, Milan, Italy). Osteogenic medium was changed two times/week. Lymphoblastoid cell lines (LCLs) were obtained by infecting peripheral blood lymphocytes (PBL) with Epstein–Barr Virus (EBV).

### 4.2. Proliferation Assay

Cells were seeded at a density of 4 × 10^4^ in 6-well plates, trypsinized at different time points (1, 4, and 8 days), and counted in a Bürker chamber after addition of Trypan Blue (Sigma-Aldrich, Milan, Italy).

### 4.3. Alizarin Red Staining

Osteoblasts fixed in 75% ethanol were stained for 10 min with Alizarin Red solution and then thoroughly washed with deionized water. The staining was quantified by measuring the absorbance with the plate reader Infinite 200 (Tecan Group Ltd., Männedorf, Switzerland). Alizarin Red binds to calcium present in the extracellular matrix deposited by osteoblasts, and therefore its positive staining is associated with the amount of calcium present [43].

### 4.4. Immunofluorescence Staining

Cells were fixed in 1% paraformaldehyde in PBS and immunostained with a rabbit anti-SBDS antibody (1:50, Abcam plc, Cambridge, UK) for 3 h at room temperature, followed by incubation with a 488-Alexa Fluor-conjugated anti-rabbit secondary antibody (1:500, Thermo Fisher Scientific Inc., Waltham, MA, USA). DAPI (1 µg/mL, Thermo Fisher Scientific Inc., Waltham, MA, USA) was used for nuclear labeling. For actin labeling, 555-Alexa Fluor-conjugated phalloidin (1:40, Thermo Fisher Scientific Inc., Waltham, MA, USA) was used. Secondary antibodies were incubated for 45 min protected from light. Cover slips were mounted with FluoromountG (Southern Biotech, Birmingham, AL, USA). Images were acquired using the Nikon Eclipse 50i, 40X objective.

### 4.5. RNA Extraction, Reverse Transcription, and RT-qPCR

RNA was extracted from a second-passage confluent osteoblast culture using RNeasy Plus kit (Qiagen Germantown, MD, USA). RNA was quantified using an ND-1000 UV-Vis Spectrophotometer (Thermo Scientific, Wilmington, DE, USA), and the integrity of the RNA was assessed with an Agilent 2100 Bioanalyzer (Agilent Technologies Inc., Lexington, MA, USA) according to the manufacturer’s instructions. All the RNA samples used in this study exhibited a 260/280 ratio above 1.9 and an RNA integrity number (RIN) above 9.0. An amount of 500 ng of total RNA was reverse transcribed to cDNA using oligo(dT) and random primers and RNase H+ and MMLV reverse transcriptase (Bio-Rad, Hercules, CA, USA iScript Reverse Transcription kit,). cDNAs were subjected to RT-qPCR reactions. The relative expression of *SBDS* mRNA was evaluated using the following specific primers:

*SBDS_*FW AGATAGAACGTGCTCACATGAGGC;
*SBDS*_REV GGTGTCATTCAAATTTCTCATGTC;
*β-ACT*_FW CATGTACGTTGCTATCCAGGC;
*β-ACT*_REV CTCCTTAATGTCACGCACGAT,

while the analysis of osteogenesis-related genes, namely, runt-related transcription factor 2 (*RUNX2*), osterix (*OSX*), osteopontin (*OPN*), bone sialoprotein (*BSP*), osteocalcin (*BGLAP*), alkaline phosphatase (*ALP*), and collagen type I (*COL1A*) mRNA, was performed using primer–probe sets validated and purchased as “Assay-on-Demand” (Thermo Fisher Scientific Inc., Waltham, MA USA) in a singleplex PCR mix. The RT-qPCR reaction was performed in an ABI PRISM^®^ 7900 Sequence Detection System (Thermo Fisher Scientific Inc., Waltham, MA, USA). Each gene expression was first normalized with *β-actin* content, and the relative quantification was calculated with the 2^−ΔCt^ method. Three replicates were performed for each experimental point, and experiments were repeated with cells obtained from different donors.

To analyze the alternative *SBDS* transcript, RNA was extracted from lymphoblastoid cell lines and osteoblast cell lines of a healthy control, an SDS patient (SDS3) carrying the mutation 258 + 2T > C in the heterozygous state, and an SDS patient (SDS9) homozygous for the mutation 258 + 2T > C. PCR reactions were performed using the following specific primers:

g*SBDS_*FW GGAACAGATGACCAAACTGAAATC;
g*SBDS_*REV TCAATAAGGATCACGGTGTATGG,

at the following condition: 94 °C for 30 s, 94 °C for 10 s, 62 °C for 15 s, and 72 °C for 20 s for 35 cycles, and a final step of 72 °C for 7 min 30 s. For the PCR reaction, 25 ng of WT LCL and human OB cDNA, 50 ng of SDS3 LCL and human OB cDNA, and 250 ng of SDS9 LCL and human OB cDNA were used. The RT-PCR products were run on agarose gel at 5% using *GellyPhor* ULTRA agarose (Euroclone srl, Milan, Italy).

### 4.6. One-Color Expression Arrays

Microarray expression profiling was performed on second-passage confluent osteoblasts derived from SDS patients (*n* = 3) and from healthy subjects (*n* = 3) cultured in basal medium (IMDM with 10% FBS). Microarray expression profiling was carried out according to Agilent’s One-Color Microarray-Based Gene Expression Analysis Low Input Quick Amp Labeling Protocol (Version 6.9.1) using Low Input Quick Amp Labeling Kit, One-ColorAgilent, and Agilent Whole Transcriptome (WT) Oligo Human Microarray slides 8 × 60K format (Agilent Technologies, Santa Clara, CA, USA). Data analysis was performed using Agilent GeneSpring 14.9.1 software. Data from each sample were imported into the software with the following parameters: Threshold: 1; Logbase: 2; Normalization: Shift to 75.0 percentile; Baseline Transformation: median of all samples. Clustering analysis was performed by hierarchical analysis on normalized intensity values with Euclidean distance metrics and Ward’s linkage rules both on all genes and on selected gene sets.

### 4.7. Western Blot Analysis

After removing the medium, adherent cells were gently scraped in 75 µL modified RIPA buffer (EDTA 10 mM) with a protease and phosphatase inhibitor cocktail (1:50 and 1:100, Sigma-Aldrich, Milan, Italy). The lysates were centrifuged at 12,000 rpm for 10 min at 4 °C, supernatants were collected, and the total protein concentration was determined by BCA assay (Pierce, Rockford, IL, USA). An amount of 30 µg of total protein extract was mixed with the appropriate volume of denaturing Laemmli sample loading buffer, heated at 100 °C for 5 min, and loaded onto 4–15% Tris-Gly precast polyacrylamide gels (Bio-Rad, Hercules, CA, USA). Western blots were performed using specific antibodies against human SBDS (1:300), p53 (1:300), β-actin (1:2000), and ALP (1:500), which were purchased from Santa Cruz Biotechnology Inc., Heidelberg, Germany. BSP (1:1000), OPN (1:500), and OSX (1:1000) were purchased from Immunological Sciences, Rome, Italy. RUNX2 (1:500) and cleaved/full length Caspase 3 antibody (1:500) were purchased from Cell Signaling Technology, Danvers, MA, USA. Antibodies were diluted in 5% milk or BSA Tris-buffered saline with 0.1% Tween20 (Sigma-Aldrich, P9416). After washing, membranes were treated with specific horseradish peroxidase-conjugated secondary antibodies (1:2000). Bound peroxidase activity was revealed using the enhanced chemiluminescence substrate (Pierce, Rockford, IL, USA). The signal was acquired with the UVITEC MiniHD9 (Cambridge, UK) and quantified by means of the manufacturer’s Nine Alliance software v. 17.01. β-actin was used as a loading control.

### 4.8. Tp53 Mutation Analysis

Total RNA was extracted from 11 SDS-hOBs and 4 H-hOBs, quantified, and retrotranscribed, as described in Section 4.5. The primers for the PCR reaction were designed to cover the entire *Tp53* coding region (NM_000546.6). PCR reactions were performed using the following primers:

p53 _93FW: GTGACACGCTTCCCTGGATT;
p53_962REV: CACGCACCTCAAAGCTGTTC;
p53_569FW: GTGCAGCTGTGGGTTGATT;
p53_1067REV: GCAGTGCTCGCTTAGTGCTC;
p53_1355REV: GCTGTCAGTGGGGAACAAGAA,

at the following condition: 94 °C for 30 s, 94 °C for 30 s, 58 °C for 30 s, and 72 °C for 30 s for 35 cycles, and a final step of 72 °C for 4 min 30 s. The amplicons were purified using DNA Clean & Concentrator Kits (Zymo Research, Irvine, CA, USA). All the samples were analyzed by direct Sanger sequencing.

### 4.9. siRNA-Mediated p53 Gene Silencing

Sub-confluent (80%) SDS-OBs were transfected either with a short interfering RNA (siRNA) targeting human p53 (Hs_TP53_9 FlexiTube siRNA, functionally verified siRNA directed against human *Tp53*, final concentration 5 nM; Qiagen, Milan, Italy) or a scrambled control (AllStars Negative Control, Qiagen, Milan, Italy). The transfection was performed using the HiPerFect transfection reagent (Qiagen, Milan, Italy) according to the manufacturer’s instructions. In the osteogenic differentiation experiments, siRNAs were added twice a week.

### 4.10. Statistical Analysis

Statistical analysis was performed with Prism vs. 5.04, (GraphPad Software, San Diego, CA, USA). Statistically significant differences were determined using a nonparametric ANOVA test for repeated measures (Friedman test) followed by a multiple comparisons test (Dunn’s post-test), by the Mann–Whitney test, or by the Wilcoxon test. Up- and downregulated genes in the array results were extrapolated by applying a moderated *t*-test, followed by Benjamini–Hochberg *p*-value correction, with a threshold of *p* = 0.05 and fold change cut-off of >2.0, to the SDS patient-derived osteoblasts vs. healthy subject-derived osteoblasts.

## Figures and Tables

**Table 1:** Genetic features of the patients with Shwachman–Diamond syndrome.

ID	*SBDS* Mutations (m = Maternal; p = Paternal; u = Undetermined)
SDS1	c.183_184TA > CT, p.K62X (m); c.258 + 2T > C, p.C84fsX3 (p)
SDS2	c.183_184TA > CT, p.K62X (u); c.258 + 2T > C, p.C84fsX3 (u)
SDS3	c.183_184TA >CT, p.K62X (m); c.258 + 2T > C, p.C84fsX3 (p)
SDS5	c.183_184TA > CT, p.K62X (m); c.258 + 2T > C, p.C84fsX3 (p)
SDS6	c.258 + 2T > C, p.C84fsX3 (m); c.183_184TA > CT, p.K62X (p)
SDS7	c.258 + 2T > C, p.C84fsX3 (m); c.183_184TA > CT, p.K62X (p)
SDS8	c.258 + 2T > C, p.C84fsX3 (u); c.92_93GC > AG, p.C31X (u)
SDS9	HOMO c.258 + 2T > C, p.C84fsX3 (m;p)
SDS10	c.258 + 2T > C, p.C84fsX3 (m); c.183_184TA > CT, p.K62X (p) c.258 + 2T > C, p.C84fsX3 (de novo mutation)
SDS11	c.356G > A, p.V57I (m); c.258 + 2T > C, p.C84fsX3 (p)
SDS12	c.258 + 2T > C p.C84fsX3 (m); c.183_184TA > CT p.K62X(p); c.258 + 2T > C, p.C84fsX3 (p)
SDS13	c.258 + 2T > C, p.C84fsX3 (m); c.289_292delGATA (C139X) (p)
SDS14	c.258 + 2T > C, p.C84fsX3 (m); c.183_184TA > C, p.K62X(p)

## Data Availability

Microarray data are available at https://www.ebi.ac.uk/arrayexpress/; accession number: E-MTAB-9397.

## Supplementary Materials

The following are available online at https://www.mdpi.com/article/10.3390/ijms222413331/s1.
