# Supplementary material for: Enhanced p53 Levels Are Involved in the Reduced Mineralization Capacity of Osteoblasts Derived from Shwachman–Diamond Syndrome Subjects
Source: Int J Mol Sci. 2021 Dec 11;22(24):13331. doi: 10.3390/ijms222413331 (PMC8707819; doi:10.3390/ijms222413331)
Supplement: Supplementary file 1 [file ijms-22-13331-s001.zip › Suppl.Figures.pdf]

Figure S1

a

b

c

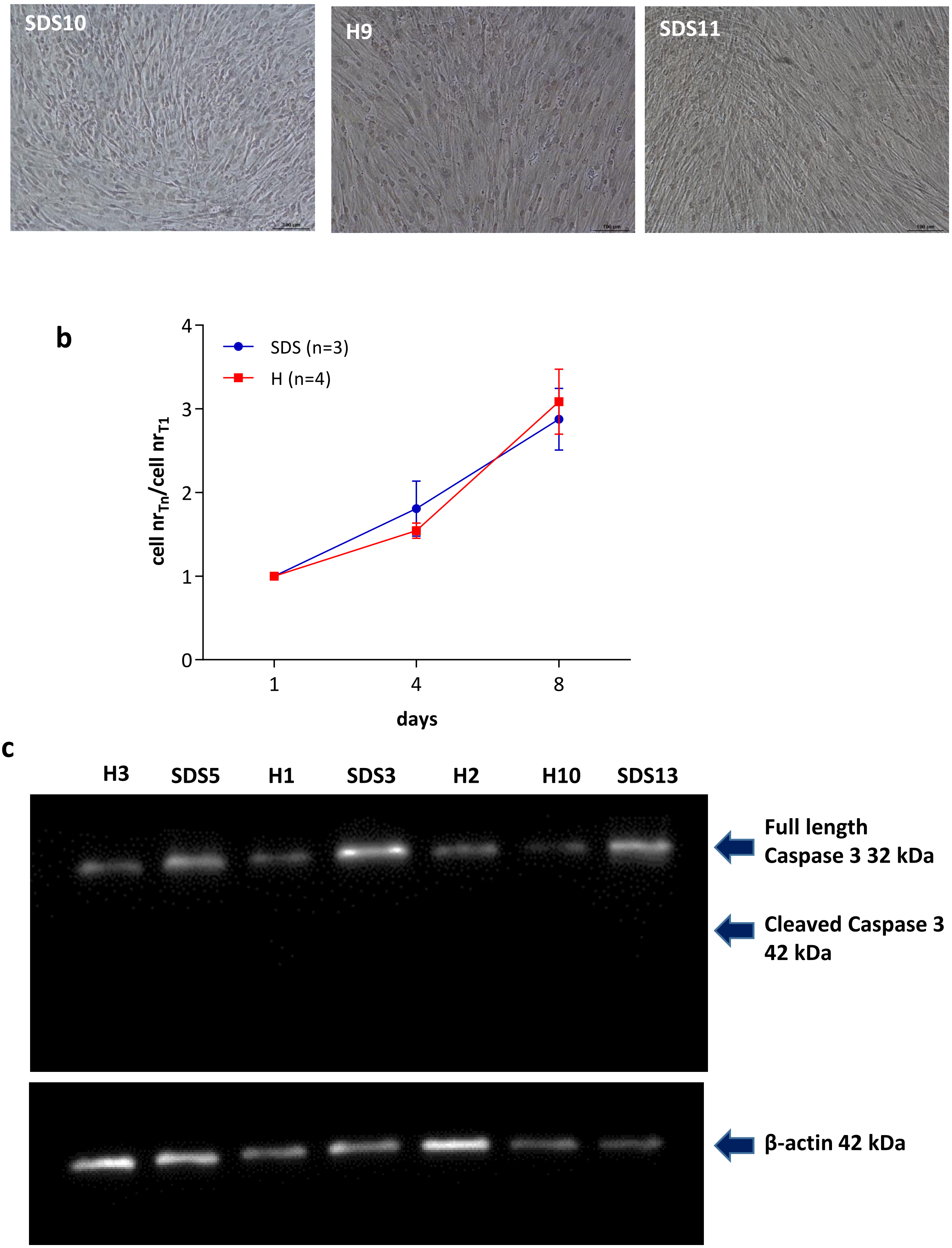

d

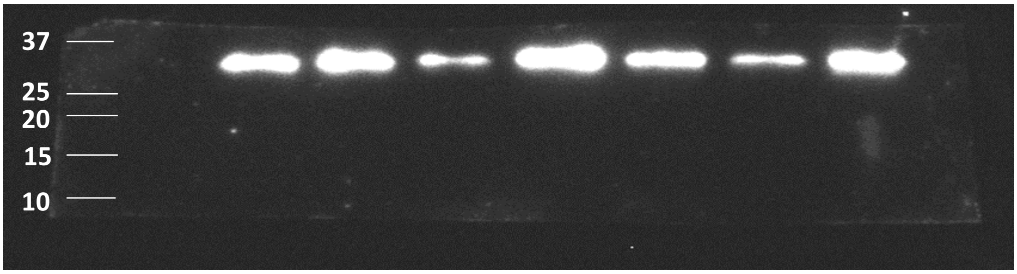

e

Antibody #9668 Cell Signaling Technologies.
Western blot analysis of HeLa cell extracts treated with 1uM staurosporine (3hrs)

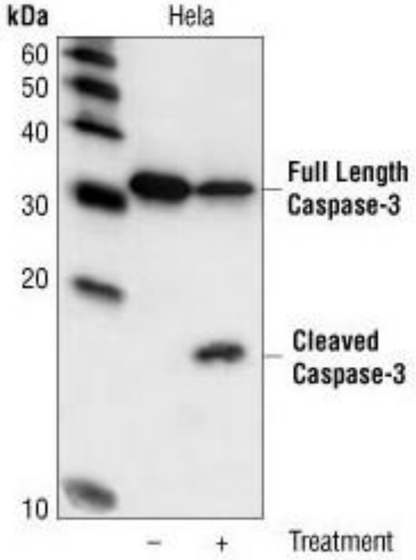

Figure S1: a. Image of osteoblast derived from Shwachman Diamond Syndrome subjects (SDS10, SDS11) and from a healthy subject (H9), magnification 10x. **b**. Proliferation of osteoblast derived from Shwachman Diamond Syndrome subjects (SDS=3) and from healthy subjects (n=4). **c**. Representative image of Western blot analysis of full length and cleaved caspase 3 protein expression in osteoblasts from healthy subjects (H3,H1. H2) and SDS patients (SDS5,SDS3,SDS13) **d.** Over-exposed image of the western blot did not reveal the presence of cleaved caspase 3 **e**. Characteristics of the antibody used for the detection of full length and cleaved caspase. all these data indicate that SDS-OBs has comparable proliferation and apoptotic activies as H-OBs.

Figure S2

(a)

```
>Human SBDS chromosome:GRCh37:7:66452064:66461188
   1 aattagcttggcgtggccgctcacacctgtagtcccagctacttgggaagctaacgctgg    60
  61 aagatcgctcgaacccaggaggtcgaagctgcagtggggccatgaccctgccactgcact   120
 121 ccagcctgggtgacagagtgagactgacttaaaaaaaaaactgtaaataaaaaagcaaca   180
 181 aaaaaatgaaaaaagaataaaacaaggatggcaaaatgttgatagcggggtgatgggtac   240
 241 atagaaggttcattacactttttgtctacattttatgttaaaatatttctattataaaa   300
 301 gggtgagtaaataaaaatatttattataaaataattctattataaaaaatatttatatta   360
 361 taaagtgggtctggcccttgaattccgcggaacgaggtggtgccaacgctgtgttttaac   420
 421 ccggtcactaaacatccgcgagcatcctgtcagagctctcagctcattggcgaaagtaaa   480
 481 tacgccaaggaaaagcacctccctttttgggcgtggaaagatggcgtaaaaagccacaat   540
 541 acgcaggcgtcatcgctcacttttcccctcccggcttctgctccacctgacgcctgcgca   600
 601 GTAAGTAAGCCTGCCAGACACACTGTGACGGCTGCCTGAAGCTAGTGAGTCGCGGCGCCG   660  exon 1
 661 CGCACTGGTGGTTGGGTCAGTGCCGCGCGCCGATCGGTCGTTACCGCGAGGCGCTGGTGG   720
 721 CCTTCAGGCTGGACGGCGCGGGTCAGCCCTGGTTCGCCGGCTTCTGGGTCTTTGAACAGC   780
 781 CGCGATGTCGATCTTCACCCCCACCAACCAGATCCGCCTAACCAATGTGGCCGTGGTACG   840
 841 GATGAAGCGTGCCGGGAAGCGCTTCGAAATCGCCTGCTACAAAAACAAGGTCGTCGGCTG   900
 901 GCGGAGCGGCGTgtgagtagccccctccctcgggcctgggcctgggcctgagccgtcacC   960
 961 tccgaggcggcctgtctctgcccaagtcgagtgaatgggccaggctggggtgttggccgg  1020
1021 ggaggaaatggaacattcctgctgtgagcatgagacgtcgctgtccgagcttggcgccta  1080
1081 agccaagggtttcttctttatttggttggttcggattgggttgttggtttggggttttgt  1140
1141 tttgttggtgtcataaaagctgcagccaagaaatctcgtaattgtggtccttttcctaga  1200
1201 ataatgatggctgagaacctagtcttacgaatactgtcatagttgtgggtacatctggtg  1260
1261 tcatagatctcagtagccctagcagtttaaagtgcgtagtgtcttcactgctgccatcta  1320
1321 gggactggcattccgtggcagcagctgtctgcaccccaccccacccttgtgtttcttacg  1380
1381 tcctcccatgacatttttctccagtgagcaaatggtaaggcaaatacggttctgagtttt  1440
1441 gaaaatgttccctcaggccgatgcgggcagttcacttgaggccaggagttcgaggccagc  1500
1501 ctggccaacatgaaaccccatctctactaaaaatacaaagttagccgggtgtggtggcgc  1560
1561 atgcctgtaatcccagttactcaggaggctgaggcgggagaatcacttgaacccgggagg  1620
1621 ctgaggttacagtgacccgagatcgcgccattgcactccagcctgggcaaaaacagtgaa  1680
1681 attccatctaggggcggggggttggggggtaagaaaaagaaaactgccctctacactaaag  1740
1741 gtcatcaggggggatttgttgtgtcttgccgttcatgttgttgccatctcgtatttaaatg  1800
1801 taaatgcatgtccaagtttcaagtatattcacataggactttctctcctgccctcacaag  1860
1861 GGAAAAAGACCTCGATGAAGTTCTGCAGACCCACTCAGTGTTTGTAAATGTTTCTAAAGG  1920  exon 2
1921 TCAGGTTGCCAAAAAGGAAGATCTCATCAGTGCGTTTGGAACAGATGACCAAACTGAAAT  1980
         ▼         ▼c.258+2T>C
1981 CTGTAAGCAGgtgggtaacagctgcagcatagctaaccctaataaccatttataacgtat  2040
       └── del 8 bp ──┘
2041 ttgtagatatattaaacattaaaggctgttttctggaggaaagactaaccaagcaataa  2100
2101 tgtgaactgcacagtgtcacttctaataataaagaacttggtggttttgtttgtttttttg  2160
2161 tggtttttttttttttttttttgccaaaacctcctgaaatcagattttctactaaattac  2220
2221 ctcatttttctataaattgccccatttatagaatgtggtatacctgaaaactgaatttgt  2280
2281 tttcaagtatttcatctttcagatcctttttttttttgggtgggggacagagtcttgctc  2340
2341 tgttgcccaggctggagtgcagtgcacttcactgcactcagctcactgcaacctctgcct  2400
2401 cctggattcaagcgattctcttgcctcagcttccagggtagctgggattacaggtgccca  2460
2461 ccaccctgcctggctaattttggattttagtagagatggggtttcaccatgttggcca  2520
2521 ggttggtctcaaactcctgaccttgggtgatatgcccgccttagccacccaaagtgctgg  2580
2581 gattacagatgtgagccactgagcccagccaaaagattcttgcatcttttgggcaaagct  2640
2641 caaaccattacttacatattgatagctggagaggatgaaatttaatttctctccatcca  2700
2701 gttactcatttttatggttagttaataaatagtgtgtgatagagaaagatagtgatttc  2760
2761 ttaaatgtgttggcatttttttagATTTGACTAAAGGAGAAGTTCAAGTATCAGATAAA  2820  exon3
2821 GAAAGACACACACAACTGGAGCAGATGTTTAGGGACATTGCAACTATTGTGGCAGACAAA  2880
2881 TGTGTGAATCCTGAAACAAAGAGACCATACACCGTGATCCTTATTGAGAGAGCCATGAAG  2940
2941 GACATCCACTATTCGGTGAAAACCAACAAGAGTACAAAACAGCAGgtgagtggtttctca  3000
```

(b)

```
WT           ATGTCGATCTTCACCCCCACCAACCAGATCCGCCTAACCAATGTGGCCGTGGTACGGATG exon1
258+2T>C     ATGTCGATCTTCACCCCCACCAACCAGATCCGCCTAACCAATGTGGCCGTGGTACGGATG
WT pep        M  S  I  F  T  P  T  N  Q  I  R  L  T  N  V  A  V  V  R  M
Alt pep       M  S  I  F  T  P  T  N  Q  I  R  L  T  N  V  A  V  V  R  M

WT           AAGCGTGCCGGGAAGCGCTTCGAAATCGCCTGCTACAAAAACAAGGTCGTCGGCTGGCGG
258+2T>C     AAGCGTGCCGGGAAGCGCTTCGAAATCGCCTGCTACAAAAACAAGGTCGTCGGCTGGCGG
Wt pep        K  R  A  G  K  R  F  E  I  A  C  Y  K  N  K  V  V  G  W  R

Alt pep       K  R  A  G  K  R  F  E  I  A  C  Y  K  N  K  V  V  G  W  R

WT           AGCGGCGTGGAAAAAGACCTCGATGAAGTTCTGCAGACCCACTCAGTGTTTGTAAATGTT exon2
258+2T>C     AGCGGCGTGGAAAAAGACCTCGATGAAGTTCTGCAGACCCACTCAGTGTTTGTAAATGTT
WT pep        S  G  V  E  K  D  L  D  E  V  L  Q  T  H  S  V  F  V  N  V
Alt pep       S  G  V  E  K  D  L  D  E  V  L  Q  T  H  S  V  F  V  N  V

WT           TCTAAAGGTCAGGTTGCCAAAAAGGAAGATCTCATCAGTGCGTTTGGAACAGATGACCAA
258+2T>C     TCTAAAGGTCAGGTTGCCAAAAAGGAAGATCTCATCAGTGCGTTTGGAACAGATGACCAA forw ►
WT pep        S  K  G  Q  V  A  K  K  E  D  L  I  S  A  F  G  T  D  D  Q
Alt pep       S  K  G  Q  V  A  K  K  E  D  L  I  S  A  F  G  T  D  D  Q

WT           ACTGAAATCTGTAAGCAGATTTTGACTAAAGGAGAAGTTCAAGTATCAGATAAAGAAAGA  exon3
258+2T>C     ACTGAAATCT--------ATTTTGACTAAAGGAGAAGTTCAAGTATCAGATAAAGAAAGA
WT pep        T  E  I  C  K  Q  I  L  T  K  G  E  V  Q  V  S  D  K  E  R
Alt pep       T  E  I           Y  F  D  X

WT           CACACACAACTGGAGCAGATGTTTAGGGACATTGCAACTATTGTGGCAGACAAATGTGTG
258+2T>      CACACACAACTGGAGCAGATGTTTAGGGACATTGCAACTATTGTGGCAGACAAATGTGTG
WT pep        H--T--Q--L--E--Q--M--F--R--D--I--A--T--I--V--A--D--K--C--V-

WT           AATCCTGAAACAAAGAGACCATACACCGTGATCCTTATTGAGAGAGCCATGAAGGACATC
258+2T>C     AATCCTGAAACAAAGAGACCATACACCGTGATCCTTATTGAGAGAGCCATGAAGGACATC ◄ rev
WT pep        N--P--E--T--K--R--P--Y--T--V--I--L--I--E--R--A--M--K--D--I
```

Figure S2. Splice site mutation in the *SBDS* gene
(a) Genomic sequence of the gene *SBDS* showing the first 3 exons (capital letters) and introns (lowercase letters). It is indicate the position of the mutation c.258+2T>C (▼)that abrogates the classical 3’ splice site GT and creates the mutated and weak splice site GC, and the position (▼)of the alternative splice site used in the alternative transcript, resulting in the deletion of 8 nucleotides.
(b) The alignment of wild type (WT), mutated (258+2T>C) nucleotide sequences and the amino acids sequence of the alternative transcript (alt pep) and the wild type protein at the mutation level (WT pep). Primer sequences (forward and reverse) used for the RT-PCR are underlined and in bold.
